# Soft Tissue Tumours of the Retroperitoneum

**DOI:** 10.1155/S1357714X00000049

**Published:** 2000-03

**Authors:** J. Frans Graadt Van Roggen, Pancras C. W. Hogendoorn

**Affiliations:** 1 Department of Pathology Leiden University Medical Centre The Netherlands

## Abstract

*Purpose.* This review summarizes the more prevalent soft tissue tumours arising in the retroperitoneum and highlights some recent fundamental and diagnostic developments relevant to mesenchymal tumours.

*Discussion.* The retroperitoneum is an underestimated site for benign and malignant neoplastic disease, and represents the second most common site of origin of primary malignant soft tissue tumours (sarcomas) after the deep tissues of the lower extremity. In contrast to the predominance of benign soft tissue lesions over malignant sarcomas elsewhere, retroperitoneal mesenchymal lesions are far more likely to be malignant. The differential diagnosis is primarily with the more common lymphoproliferative and parenchymatous epithelial lesions arising in this area, and with metastatic disease from known or unknown primary sites elsewhere.The most prevalent mesenchymal tumours at this site are of a lipomatous, myogenic or neural nature.Their generally late clinical presentation and poorly accessible location provides numerous clinical challenges; optimal radiological imaging and a properly performed biopsy are essential cogs in the management route. Histopathological diagnosis may be complicated, but has been aided by developments in the fields of immunohistochemistry and tumour (cyto)genetics. Despite significant advances in oncological management protocols, the prognosis remains generally less favourable than for similar tumours at more accessible sites.


Sarcoma (2000) 4, 17–26

ORIGINAL ARTICLE

### Soft tissue tumours of the retroperitoneum

J. FRANS GRAADT VAN ROGGEN & PANCRAS C.W. HOGENDOORN

*Department of Pathology, Leiden University Medical Centre, The Netherlands*

**Abstract**

*Purpose.* This review summarizes the more prevalent soft tissue tumours arising in the retroperitoneum and highlights some recent fundamental and diagnostic developments relevant to mesenchymal tumours.

*Discussion.* The retroperitoneum is an underestimated site for benign and malignant neoplastic disease, and represents the second most common site of origin of primary malignant soft tissue tumours (sarcomas) after the deep tissues of the lower extremity. In contrast to the predominance of benign soft tissue lesions over malignant sarcomas elsewhere, retroperitoneal mesenchymal lesions are far more likely to be malignant. The differential diagnosis is primarily with the more common lymphoproliferative and parenchymatous epithelial lesions arising in this area, and with metastatic disease from known or unknown primary sites elsewhere. The most prevalent mesenchymal tumours at this site are of a lipomatous, myogenic or neural nature. Their generally late clinical presentation and poorly accessible location provides numerous clinical challenges; optimal radiological imaging and a properly performed biopsy are essential cogs in the management route. Histopathological diagnosis may be complicated, but has been aided by developments in the fields of immunohistochemistry and tumour (cyto)genetics. Despite significant advances in oncological management protocols, the prognosis remains generally less favourable than for similar tumours at more accessible sites.

**Key words:** *soft tissue tumour, sarcoma, review, retroperitoneum*

### Introduction

The retroperitoneum represents the lumbo-iliac anatomical region bounded anteriorly by the peritoneum, posteriorly by the posterior abdominal wall, superiorly by the 12th rib and vertebra, inferiorly by the sacrum and iliac crest and laterally by the peripheral margin of the quadratus lumborum muscles. Present within the retroperitoneum are the pancreas and duodenum, kidneys and ureters, adrenal glands, aorta and its branches, inferior vena cava and its tributaries, lymph nodes and nerve branches, all embedded in a loose framework of connective tissue.

The relative paucity of vital structures, and the abundance of loose connective tissue in this area, results in a generally late clinical presentation of space-occupying lesions. Symptoms tend to be related to gastrointestinal, urinary or vascular compromise, when large lesional size and compression/invasion of adjacent structures severely limits the curative treatment options.

Collectively, malignant tumours of the retroperitoneum are roughly four times more frequent than benign lesions, in sharp contrast to neoplastic disease occurring elsewhere in the body, where benign disease predominates.[1,2]

In adults the majority of retroperitoneal neoplasms are primary lymphoproliferative (Hodgkin's and non-Hodgkin's lymphoma) and parenchymatous epithelial tumours (renal, adrenal, pancreas), or represent metastatic disease from known or unknown primary sites elsewhere.[2] In infancy and early childhood, nephroblastoma (Wilms' tumour), neuroblastoma and germ cell tumours are the more common retroperitoneal neoplasms.[3]

Soft tissue (mesenchymal) tumours of the retroperitoneum are less common; nevertheless 15% of all primary sarcomas arise within the retroperitoneum, and consequently this represents the second most common site for the origin of malignant mesenchymal tumours, after the deep tissues of the lower extremity (thigh).[1] It is the general experience that, although benign soft tissue lesions at all sites outnumber their malignant counterparts by a ratio of at least 100:1, in the retroperitoneum sarcomas are more prevalent than their benign counterparts. In comparison with soft tissue lesions elsewhere, however, the range of commonly encountered retroperitoneal mesenchymal neoplasms is more limited, with the most common lesions being of a lipomatous, smooth muscle or neural nature (Table 1).[1,2]

Correspondence to: P.C.W. Hogendoorn, MD, PhD, Department of Pathology, Leiden University Medical Centre, Building I, L1-Q, PO Box 9600, 2300RC, Leiden, The Netherlands. Tel: +31 71 526 6639; Fax: +31 71 5248158; E-mail: p.c.w.hogendoorn@pathology.medfac.leidenuniv.nl

1357-714Xprint/1369-1643online/00/010017-10 © 2000 Taylor & Francis Ltd

**Table 1**. *Soft tissue neoplasms of the retroperitoneum*

| Malignant | Benign |
|---|---|
| Lipomatous: | |
| Well-differentiated liposarcoma | Myolipoma |
| Dedifferentiated liposarcoma | |
| Myogenic: | |
| Leiomyosarcoma | |
| Neurogenic: | |
| Malignant peripheral nerve sheath tumour | (Anciant) schwannoma |
| Miscellaneous: | Neurofibroma |
| Desmoplastic small round cell tumour | |
| Pleomorphic sarcoma, undifferentiated | |
| Kaposiform haemangioendothelioma | |
| Inflammatory myofibroblastic tumour | |

While most of the better differentiated retroperitoneal lesions generally do not pose significant diagnostic problems, high-grade sarcomas may present morphologically as poorly differentiated tumours lacking distinctive phenotypic features. Consequently, histomorphological and ultrastructural analysis may be insufficient for reaching an accurate (differential) diagnosis in certain cases, essential for directing patient management strategies. Fortunately, it has become apparent over the last decade that certain soft tissue tumours appear to be associated with tumour-specific genetic alterations (Table 2), and (cyto)genetic analysis aimed at the detection of these alterations is a helpful adjunct in resolving some of these diagnostic dilemmas.[4]

Computerized tomography (CT) scanning and (dynamic) magnetic resonance imaging (MRI) are the radiological procedures of choice in determining the extent of local, regional and distant tumour spread. These procedures are essential for clinical staging, planning an appropriate and optimal biopsy procedure prior to definitive treatment, as well as playing an important role in follow-up imaging to assess efficacy of treatment regimens.[5–7] Additionally, positron emission tomography (PET) scanning may, in certain settings, be of clinical use, particularly in the detection of high-grade sarcomas and the assessment of local recurrence.[8,9]

It is the aim of this short synopsis to discuss the most prevalent retroperitoneal soft tissue tumours and associated differential diagnoses, and to highlight some important recent developments in fundamental and diagnostic aspects of mesenchymal tumours.

### The histopathology of retroperitoneal soft tissue tumours

The soft tissues are defined as consisting primarily of extraparenchymatous voluntary muscle, fat, fibrous (connective) tissue, and the neurovascular supply of these tissues.[1] Nevertheless, mesenchymal tumours also regularly arise from the supporting connective tissues of the various organs and are included in this review.

### *Reactive non-neoplastic processes*

Retroperitoneal masses may be of a non-neoplastic or neoplastic nature. Non-neoplastic masses arising at this site are predominantly of a reactive/inflammatory or infectious nature, involving either the organs at this site or the retroperitoneal soft tissues. These generally do not pose a clinical problem, although periodically the differential diagnosis with neoplastic disease may be more complicated; for example the differential diagnosis of reactive fibrosis

**Table 2**. *Genetic alterations of diagnostic significance in the sarcoma group*

| Tumour | Genetic alteration | Gene(s) |
|---|---|---|
| Alveolar rhabdomyosarcoma | t(2; 13)(q35; q14) | *FKHR/PAX3* |
| | t(1; 13)(q36; q14) | *FKHR/PAX7* |
| Atypical lipoma/well-differentiated liposarcoma | ring (chr. 12)/marker chromosome | ? |
| Desmoplastic small round cell tumour | t(11; 22)(p13; q12) | *EWS/WT1* |
| Ewing's sarcoma/PNET | t(11; 22)(q24; q12) | *EWS/FLI1* |
| | t(21; 22)(q22; q12) | *EWS/ERG* |
| | t(7; 22)(p22; q12) | *EWS/ETV1* |
| Gastrointestinal stromal tumour | mutation chromosome 4q11-21 | *c-kit* |
| | losses chromosome 14q | |
| Myxoid liposarcoma | t(12; 16)(q13; p11) | *CHOP/FUS* |
| | t(12; 22)(q13; q11) | *CHOP/EWS* |
| Synovial sarcoma | t(X; 18)(p11.2; q11.2) | *SYT/SSX1* |
| | | *SYT/SSX2* |

(sometimes seen with non-Hodgkin's lymphoma)[10]/ idiopathic retroperitoneal fibrosis (an inflammatory/ reactive process often presenting as a mass)[11,12]/ retroperitoneal fibromatosis,[12] at this site regularly seen in the context of Gardner's syndrome[13]/ fibrosarcoma (rare),[12] and the differential diagnosis of xanthogranulomatous pyelonephritis (inflammatory)[14]/(sarcomatoid) renal cell carcinoma[15]/soft tissue sarcoma with a xanthogranulomatous morphology.[12]

*Neoplastic disease*

As alluded to earlier, the majority of retroperitoneal tumours are primary lymphoproliferative and parenchymatous epithelial tumours.

The majority of lymphomas are non-Hodgkin's B-cell type, but these may be associated with a variable degree of contiguous fibrosis/sclerosis sometimes complicating the clinical differential diagnosis as mentioned above.[2,10]

Epithelial tumours arising in the kidneys, ureters, adrenals and pancreas may invade the retroperitoneal soft tissues. Although the various tumour subtypes may pose diagnostic problems, these tumours are histopathologically rarely difficult to distinguish from non-epithelial lesions, particularly with the use of immunohistochemistry.

Primary retroperitoneal seminomatous and non-seminomatous extra-gonadal germ cell tumours are less frequent, although teratomas occur in a relatively greater percentage of children.[16,17] Additionally embryological remnants such as tailgut cysts and other anomalies may occur in the retroperitoneal/sacral area, rarely undergoing malignant change.[18]

Metastatic disease to the retroperitoneum, from a gamut of intra- and extraperitoneal sites, represents another significant proportion of mass lesions at this site and needs to be considered in any differential diagnosis. Metastases from an undifferentiated carcinoma may create difficulties in the differential diagnosis with primary spindle cell tumours of the retroperitoneum, although this dilemma is usually resolved with an appropriate immunohistochemical panel including cytokeratins.

The majority of soft tissue tumours arising in the retroperitoneum (Table 1) are of a lipomatous, myogenic or neurogenic nature and are discussed below.

*1. Lipomatous tumours.* **Lipoma**—Myolipoma (lipoleiomyoma), a benign encapsulated lesion of variable size and composed of an admixture of mature adipocytes and bundles of smooth muscle may be encountered in the retroperitoneum.[19] Nevertheless, pure retroperitoneal lipomas are a poorly documented and contentious entity[20–22] and, although they theoretically must occur, all lipomatous tumours at this site should probably be regarded with a high level of suspicion for malignancy.

**Liposarcoma**—Liposarcomas are the most common soft tissue tumours occurring within the retroperitoneum, and probably account for 25–35% of all mesenchymal lesions at this site.[21–23] Although the liposarcoma group is subdivided into well-differentiated liposarcoma, dedifferentiated liposarcoma, myxoid/round cell liposarcoma, and pleomorphic liposarcoma, the majority of retroperitoneal liposarcomas are of the well-differentiated and dedifferentiated type.[22] Myxoid/round cell liposarcoma occur far less frequently in the retroperitoneum, while pleomorphic liposarcoma at this site is very rare.[22] Well-differentiated and dedifferentiated liposarcoma should probably be regarded as related entities since the latter often arises from/within the former,[23–25] and common genetic alterations are present in these tumours (ring chromosomes derived from chromosome 12 and large marker chromosomes; Table 2).[26,27] Similarly (cyto)genetic analysis has established that the vast majority (75%) of myxoid and round cell liposarcomas share a common t(12; 16)(q13; p11) translocation (Table 2), indicating that these tumours probably represent the low-grade and high-grade components respectively of a single tumour entity.[4,22,27] Furthermore, myxoid liposarcoma has also been reported to undergo dedifferentiation, suggesting a closer relationship between myxoid and well-differentiated liposarcoma than previously thought.[28]

Clinically, distinction between the various categories within the liposarcoma group remains important due to their different modes of biological behaviour.[22,29]

Liposarcomas may be of variable size (reaching sizes of more than 20 cm), are often well-circumscribed/encapsulated, and frequently show a distinct lobulation. The cut surface has a variable morphology dependent on tumour type.

(A) Well-differentiated and dedifferentiated liposarcoma. Well-differentiated liposarcoma is composed of a background of mature-looking adipocytes (although nuclei tend to be slightly larger and more pleomorphic), with a variable scattering of lipoblasts each containing a single atypical, hyperchromatic, scalloped (indented), and eccentrically located nucleus with one or more scattered cytoplasmic lipid droplets/ vacuoles. The tumour may be traversed by dense bands of variably cellular collagen and demonstrate a variable inflammatory cell infiltrate composed of lymphocytes and plasma cells; histopathologically the diagnosis is rarely troublesome.

Although well-differentiated liposarcomas have been subtyped (lipoma-like, inflammatory, sclerosing variants) dependent on the degree of fibrosis and inflammatory infiltrate, the clinicopathological relevance of this is debatable since combinations of the various morphologies may be seen in individual tumours.[22,30]

Dedifferentiated liposarcoma generally consists of well-differentiated areas with a sharp transition to adjacent non-lipogenic sarcomatous areas demonstrating either a low-grade fibromatosis or well-differentiated fibrosarcoma morphology, or a high-grade fibrosarcoma or undifferentiated sarcomatous morphology; occasionally the dedifferentiated areas may resemble carcinoma or melanoma.[31] Dedifferentiation to more than one histogenetic tissue type (for example osteo- or chondrosarcoma) may occur in a minority of cases.[31] The differential diagnosis of dedifferentiated liposarcoma with other low- or high-grade retroperitoneal spindle cell lesions is often difficult and may require extensive sampling of the definitive surgical specimen to identify the lipomatous component.

Well-differentiated liposarcoma is regarded as a low-grade sarcoma that does not metastasize.[23,24,32,33] However, recurrence rates are high (approaching 100% in the retroperitoneum due to the generally late presentation—and consequently large size—and problematic surgery) and a significant proportion (10–20%) will dedifferentiate over a mean period of 7–8 years with an acquisition of the capacity to metastasize, irrespective of the extent of dedifferentiation.[32] Following treatment for dedifferentiated liposarcoma, the reported rates of local recurrence and metastatic disease are variable; local recurrence occurring in 40–100% of cases and distant metastases in roughly 20% of cases.[31,34] However, the general impression remains that these tumours probably exhibit a less aggressive behaviour than dedifferentiated sarcomas in general.[31]

(B) Myxoid/round cell liposarcoma. Myxoid liposarcoma, far less common in the retroperitoneum, consists of an abundant hyaluronidase-sensitive myxoid matrix with small bland spindle-shaped or more rounded cells, univacuolated (signet ring-like) lipoblasts, and sporadic multivacuolated lipoblasts.The vasculature is composed of a delicate plexiform arrangement of thin-walled capillaries described as having a chicken wire or crow's feet distribution. The presence of a more cellular (round cell) component is associated with a more aggressive biological behaviour.[33,35,36] Since no histopathological consensus has been reached on the percentage of the round cell component required for an unambiguous diagnosis of high-grade round cell liposarcoma, we tend to report these neoplasms as mixed myxoid and round cell liposarcoma and give an approximation of the percentage round cell component present.

Depending on the extent of a round cell component, the differential diagnosis is primarily with malignant peripheral nerve sheath tumour (MPNST) and desmoplastic small round cell tumour (DSRCT).[37] This distinction is generally based on the relationship of the tumour to a large nerve trunk on imaging (MPNST), lack of coexpression of neural epithelial and mesenchymal markers (DSRCT), the degree of S100 protein positivity (spotty in MPNST, variably diffuse in liposarcoma), as well as cytomorphology. The presence of the characteristic t(12; 16), and lack of the t(11; 22)(p13; q12) translocation found in DSRCT (Table 2),[4] is a powerful adjunct.

Pure myxoid liposarcoma is regarded as a low-grade sarcoma. A metastatic potential exists, however, in about 10–20% of cases, although this correlation probably depends on the adequacy of tumour sampling (inadequate sampling will fail to identify a round cell component).[33,36] Furthermore, while a myxoid liposarcoma with a round cell component of more than 5–10% is regarded as having a significant metastatic risk, caution is necessary since no clear criteria are present for tumour grading, and there are often problems with tumour sampling.[35,36]

*2. Myogenic tumours.* **Leiomyoma**—Primary leiomyoma of the retroperitoneum is an extremely rare occurrence and should be diagnosed with extreme caution (see below).[2]

**Leiomyosarcoma**—Leiomyosarcomas of the retroperitoneum are the second most common primary malignant sarcoma at this site, after liposarcomas.[1] This tumour should not be grouped with the gastrointestinal stromal tumours (GIST) of the small and large intestine (occasionally seen in the retroperitoneum as a result of local spread), a complex group of tumours postulated to arise from pacemaker cells (interstitial cells of Cajal) located in the wall of the bowel, and demonstrating a variety of differentiation patterns (see below).[38,39]

Roughly two-thirds of leiomyosarcomas occur in women with an average age at presentation of 60 years. The tumours are of variable size (average size 10–20 cm), and at surgery often involve adjacent structures by direct spread. Tumours often show foci of haemorrhage, necrosis and cystic change.[40] Histologically, the general architecture is that of a fasicular tumour. Cell morphology can be highly variable, ranging from well-differentiated to anaplastic, but the typical cell is elongated and tapering, contains a cigar-shaped nucleus with obvious pleomorphism, bipolar perinuclear glycogen-containing vacuoles and eosinophilic cytoplasm. The detectable mitotic activity may be highly variable and, since it is the general experience that even tumours with 1–4 mitoses/2 $mm^2$ can metastasize, all tumours with obvious nuclear atypia (irrespective of the mitotic activity) should be regarded as potentially malignant when occurring in the retroperitoneum.[1] Immunohistochemically there is variable expression of muscle-specific actin (MSA), smooth muscle actin (SMA) and desmin; S100 protein and CD34 expression are absent. Although a partial deletion of chromosome 1p is present in about 50% of cases, no tumour-specific genetic alterations are consistently present, since this deletion also occurs in other soft tissue tumours.[41]

The differential diagnosis is primarily with spindle cell MPNST and fibrosarcoma, generally resolvable using immunohistochemistry (desmin positive, S100-protein negative).

These tumours are highly aggressive with a 5-year survival between 0 and 20%.[40]

**Rhabdomyosarcoma**—Rhabdomyosarcoma, although infrequently seen in the retroperitoneum, needs to be considered.[42,43] This is primarily a tumour of children and young adults and, of the three described subtypes (embryonal, alveolar and pleomorphic rhabdomyosarcoma), embryonal rhabdomyosarcoma is the most common retroperitoneal tumour, while the alveolar and pleomorphic subtypes are extremely unusual at this site. The presence of the characteristic t(2;13)(q35;q14)/t(1;13)(p36;q14) translocations present in the vast majority of alveolar rhabdomyosarcoma may be of diagnostic use[44–46] (Table 2).

*3. Neurogenic tumours.* **Schwannoma**—Schwannoma, including the cellular variant (cellular schwannoma), is the most common neurogenic soft tissue tumour occurring in the retroperitoneum.[47]

Clinically, these lesions have a broad age range of presentation, and often occur paravertebrally.

Macroscopically, both schwannoma and cellular schwannoma are encapsulated nodular tumours, regularly seen in association with a nerve trunk, which may be extremely large at presentation. On sectioning, the classical schwannoma has a variably tan, haemorrhagic and cystic appearance, while the cellular variant is firmer and more homogeneously tan in colour.

Microscopically, the schwannoma is characterized by the classical Antoni A areas composed of fasicles of spindle-shaped cells with focal nuclear palisading, hypocellular and mucoid Antoni B areas, Verocay bodies, and vascular hyalinization. The cellular schwannoma, however, as its name suggests, is more cellular, composed primarily of Antoni A areas and frequently has capsular and perivascular lymphocytic aggregates. While obvious nuclear pleomorphism and mitotic activity is generally absent in the classical schwannoma, mild nuclear pleomorphism and a variably low mitotic activity (not more than four mitoses/2 $mm^2$) may be seen in the cellular schwannoma complicating diagnosis, particularly when the pathologist receives a minute needle-core biopsy.[48,49] An accurate diagnosis is of significant clinical importance since the risks of complicated surgery at this site for what is a benign lesion should be properly considered.

The differential diagnosis is with MPNST and other spindle cell tumours, although the strong diffuse positivity for S100-protein, (peri)vascular hyalinization and Antoni A areas should in most cases facilitate the correct diagnosis.

Both tumours are benign and although local recurrences have been noted for cellular schwannoma, no metastases have been documented.[50]

**Neurofibroma**—Neurofibromas, identical to their counterparts elsewhere, are also periodically encountered in the retroperitoneum.

**Malignant peripheral nerve sheath tumour**—MPNSTs are defined as malignant mesenchymal tumours arising from, or differentiating towards, cells of the peripheral nerve sheath, but excluding epineurial tumours and tumours arising from the neural vasculature.[51] Roughly two-thirds of cases are associated with a preexisting neurofibroma, while approximately half of MPNSTs arise in patients with neurofibromatosis I (NF I) (presumably having arisen within a neurofibroma).[52] MPNSTs are tumours of adults, although the age at presentation in patients with NF I is younger than in unaffected patients.[52]

Macroscopically, tumours are nodular and firm and may show extensive necrosis. Microscopically, these tumour have a highly varied morphology (well-differentiated to anaplastic), although the prototypical lesion is cellular with a fascicular architecture; cells are elongated and tapering with wavy, pleomorphic and hyperchromatic spindled nuclei. Mitoses are frequent ($\sim$4/2 $mm^2$). Classically S100-protein staining is focal and weak to variably prominant. The presence of a rhabdomyoblastic component (malignant Triton tumour), confirmed by positive myogenic immunohistochemistry, is an extremely rare finding and may occur both within and outside the setting of NF I.[53]

MPNST is a high-grade tumour with an extremely poor prognosis ($\sim$20–30% 5-year survival).[52]

*4. Pleomorphic sarcomas.* So-called ‘malignant fibrous histiocytoma’ (MFH) is frequently regarded, probably incorrectly, as a specific diagnostic entity and used to denote a group of tumours with an anaplastic morphology and without detectable evidence of soft tissue differentiation using current immunohistochemical methods. Although this remains a controversial area, it is now likely that these tumours represent a heterogeneous group of extremely poorly differentiated tumours which are as yet not histogenetically classifiable with current techniques.[54] It is our preference to report these tumours as high-grade pleomorphic sarcomas without detectable differentiation (NOS), and to avoid the non-specific label MFH.

*5. Miscellaneous retroperitoneal soft tissue tumours.* In addition to the above-mentioned tumours, a variety of less common mesenchymal tumours are encountered in the retroperitoneum.

**Desmoplastic small round cell tumour (DSRCT)**—DSRCT, a malignant tumour of children, adolescents and young adults and belonging to the morphological category of small blue round cell tumours (including Ewing's sarcoma, embryonal rhabdomyosarcoma, lymphoma), periodically occurs at this site.[55] Histologically the tumour is composed of solid nests of small round to polygonal cells set within a dense desmoplastic stroma. Important diagnostic features are the coexpression of epithelial, neural and mesenchymal markers, and the frequent presence of a tumour-specific t(11;22)(p13;q12)

translocation involving the EWS gene on chromosome 22, and the WT1 (Wilms'tumour 1) gene on chromosome 1 (Table 2).[56]

**Gastrointestinal stromal tumours (GIST)**—The GIST category represents a set of mesenchymal tumours most commonly present in the stomach and small intestine of adults, periodically involving the retroperitoneum either by direct extension from the intra-abdominal gastrointestinal tract, or origin in the retroperitoneal portion of the duodenum.[39] This complex group of tumours are important in the differential diagnosis since they are immunohistochemically and genetically distinct from the classical leiomyoma/leiomyosarcoma, and are postulated to arise from intramural 'pacemaker cells' (interstitial cells of Cajal), demonstrating myomatous and/or neural differentiation.[39,57]

These spindle and/or epithelioid tumours generally express CD117, in contrast to smooth muscle tumours. A spectrum of biological behaviour is present, as yet difficult to predict on histomorphological basis (although a large size and a mitotic activity >5/2 $mm^2$ are pointers of aggression).[38] A proposed association with cellular Epstein–Barr virus infection has never been convincingly supported.[57] At the genetic level a proportion of these tumours are characterized by mutations in *c-kit* proto-oncogene (CD117) on chromosome 4q11-21, coding for a cell surface receptor with a tyrosine kinase function, and although this is not entirely tumour-specific it is extremely helpful in the differential diagnosis with other retroperitoneal spindle cell lesions.[39,58]

**Kaposiform hemangioendothelioma**—This is an uncommon vascular tumour occurring primarily in young children, with the retroperitoneum being one of the preferential sites of origin.[59] Macroscopically the tumour is poorly circumscribed with a grey/white appearance. Microscopically irregular cellular lobules with infiltrative margins are seen consisting of a mixture of capillariform vessels often containing microthrombi, slit-like endothelial-lined vascular spaces and spindly endothelial cells. Haemosideren deposition is variably present. Nuclear pleomorphism is mild and mitoses scarce. The differential diagnosis is with Kaposi sarcoma, a tumour rarely found in childhood, lacking a lobular architecture and containing a variable inflammatory infiltrate. Clinically the lesion does not appear to metastasize, and it is the locally invasive nature of the lesion which is responsible for the high mortality rate.

**Solitary fibrous tumour (SFT)**—SFT, a lesion of adults which can be either benign or malignant and originally described as a pleural lesion, has now been described at numerous sites including the retroperitoneum and pancreas.[60,61] Although the morphology may be highly variable, the classical examples have a 'hemangiopericytoma-like'vascular pattern and fascicles of CD34 positive spindle shaped cells intermingled with collagen bundles. The presence of significant atypia, necrosis and more than four mitoses/2 $mm^2$ are poor prognostic factors although reliable prediction of their biological behaviour is impossible. In the differential diagnosis with other spindle cell tumours, the CD34 positivity is very useful.

**Haemangiopericytoma**—Haemangiopericytoma, as a distinct clinicopathological neoplasm, remains a contentious entity and various schools of thought exist concerning this subject[62,63] Since a number of different tumours may exhibit the presumed typical 'haemangiopericytoma-like' morphology in the absence of the expected typical myoid immunohistochemical phenotype, it is probable that this may not represent a single entity but rather represent a variety of different tumours sharing a 'haemangiopericytoma-like' morphology. Nevertheless, not all tumours with a 'haemangiopericytoma-like' morphology can be readily categorized as alluded to, leaving a small subset of lesions whose true nature remains contentious.[62,63]

Paraganglioma (including a pigmented variant),[64,65] fibromatosis,[66] inflammatory myofibroblastic tumour,[67] synovial sarcoma,[68] vascular intimal sarcoma,[69] malignant mesenchymoma,[70] rhabdoid tumours,[71] extraskeletal Ewing's sarcoma[72] and extraskeletal osteosarcoma,[73,74] all described at this site, are infrequently encountered but nevertheless may enter the differential diagnosis of retroperitoneal soft tissue tumours.

### Role of fine needle aspiration/needle core biopsy

Depending on the site of the retroperitoneal lesion, fine needle aspiration/needle core biopsy may or may not be practically tenable. Fine needle aspiration (FNA), when possible, may be very useful as a first step in resolving the differential diagnosis of primary or metastatic carcinoma, a lymphoproliferative disorder, or a mesenchymal tumour. Once carcinoma and lymphoma have been excluded, its usefulness is limited since attempts at classification of mesenchymal tumours on FNA material, except in expert hands,[75] is probably unreliable and not advisable.

The role of a needle core biopsy is probably controversial. While most retroperitoneal soft tissue lesions are probably malignant (except schwannoma), and will need some form of definitive operative procedure (including schwannomas as a result of a local mass effect), the risk of tumour spill following a needle core biopsy needs to be weighed up against the possibility/need for an optimal surgical intervention.

### Tumour grading, reporting and clinical staging

Tumour grading, originally developed for soft tissue tumours located within the extremeties, represents a histological assessment based primarily on the degree

**Table 3.** *The Musculoskeletal Tumor Society staging system*

| Stage* | Grade† | Site‡ | Metastases | |
|---|---|---|---|---|
| Benign | 1 | G0 | T0 | no |
| | 2 | G0 | T1 | no |
| | 3 | G0 | T1/2 | no/yes |
| Malignant | IA | G1 | T1 | no |
| | IB | G1 | T2 | no |
| | IIA | G2 | T1 | no |
| | IIB | G2 | T2 | no |
| | IIIA/B | G1/2 | T1/2 | yes |

A frequently used staging system for benign and malignant musculoskeletal tumours of the extremeties. This system is not particularly applicable to retroperitoneal tumours where the concept of compartmentalization is a moot point.
*Stage: A=intracompartmental; B=extracompartmental.
†Grade: G0=benign; G1=low-grade malignant; G2=high-grade malignant.
‡Site: T0=intracapsular; T1=extracapsular, intracompartmental; T2=extracapsular, extracompartmental.

of tumour differentiation, mitotic activity and the extent of tumour necrosis.[76,77] In the absence of sufficient data concerning the clinical course of specific tumour types, due to their relative rarity, the intention was to try and provide an indication of the potential degree of malignancy of a lesion in order to be able to separate lesions with a good prognosis from lesions with a poor prognosis and hence to facilitate decisions with respect to the choice of treatment modalities available.[76,77]

Histological type, however, increasingly appears to be the most important parameter in determining clinical behaviour; for example synovial sarcoma is by definition a high-grade tumour irrespective of mitotic activity and extent of necrosis while in extraskeletal myxoid chondrosarcoma, patient age and tumour size appear to be the most important prognostic factors.[78] Consequently, while grading systems may broadly speaking be of clinical value, it is envisaged that increasingly tumour-specific factors will be identified having an important bearing on individual tumour behaviour.

Clinical staging of musculoskeletal tumours, via clinical examination, radiological imaging (local spread, presence of metastatic disease) and histopathological examination, serves to document the nature and extent of the disease, ideally at the time appropriate treatment is being initiated, and aims to guide therapy and provide prognostic information. While initiated by Enneking (Musculoskeletal Tumor Society staging system;[79,80] Table 3), a second (modified) staging system, designed by the American Joint Committee on Cancer (AJC) and based on the TNM system (tumour size; involvement of lymph nodes; presence of metastases) and incorporating histological tumour grade, is also in use[81] (Table 4). While the Enneking system (with its emphasis on compartmentalization) is best suited for sarcomas arising in the extremeties, the AJC system can be used for tumours at any site but is somewhat more complex.

Recently, the American Association of Directors of Anatomic and Surgical Pathology have provided recommendations for reporting common malignant tumours including soft tissue sarcomas.[82] Their aim was to provide a framework for an informative and clinically useful report to facilitate the efforts at staging and choice of optimal therapeutic regimens.

**Table 4.** *American Joint Committee on Cancer staging system*

| Stage | Grade* | Tumour† | Lymph nodes‡ | Distant metastases§ |
|---|---|---|---|---|
| Ia | G1 | T1 | N0 | M0 |
| Ib | G1 | T2 | N0 | M0 |
| IIa | G2 | T1 | N0 | M0 |
| IIb | G2 | T2 | N0 | M0 |
| IIIa | G3/4 | T1 | N0 | M0 |
| IIIb | G3/4 | T2 | N0 | M0 |
| IVa | G1–4 | T1/2 | N1 | M0 |
| IVb | G1–4 | T1/2 | N0–1 | M1 |

A widely used staging system for malignant musculoskeletal tumours, and applicable to soft tissue tumours in the retroperitoneum.
*Histological grade: G1=low (well-differentiated); G2=moderate (moderately well-differentiated); G3=high (poorly differentiated); G4=undifferentiated.
†Tumour size: T1=less than 5 cm in diameter; T2=5 cm or more in diameter.
‡ Lymph node status: N0=no lymph node metastases; N1=lymph node metastases.
§Distant metastases: M0=absent; M1=present.

### Conclusion

This brief review has highlighted the most common soft tissue tumours presenting in the retroperitoneum. Recent developments, particularly in the area of tumour genetics, are becoming increasingly useful in the diagnostic arena, and are gradually improving our knowledge about tumour histogenesis and biology. It is envisaged that a greater mechanistic understanding of tumour biology and improved diagnostic accuracy will provide optimal and tumour-specific therapeutic regimens.

### References

1 Enzinger FM, Weiss SW. *Soft tissue tumors*, 3rd edn. St Louis, MO: C.V. Mosby; 1995.
2 Rosai J. *Ackerman's surgical pathology*, 8th edn. St. Louis, MO: Mosby; 1996.
3 Crist WM, Kun LE. Common solid tumors of childhood. *N Engl J Med* 1991; 324:461–71.
4 Graadt van Roggen JF, Bovee JVMG, Morreau J, Hogendoorn PCW. Diagnostic and prognostic implications of the unfolding molecular biology of bone and soft tissue tumours. *J Clin Pathol* 1999; 52:481–9.
5 Bloem JL, Van der Woude HJ, Hogendoorn PCW. Deutsch AL, Mink JH, editors.*Bone tumors*, 2nd edn. Philadelphia: Lippincott-Raven Publishers; 1996; 11, *MRI of the musculoskeletal system: a teaching file*, pp. 655–719.
6 Verstraete KL, Van der Woude HJ, Hogendoorn PCW, de Deene Y, Kunnen M, Bloem JL. Dynamic contrast-enhanced MR imaging of musculoskeletal tumors: basic principles and clinical applications. *JMRI* 1996; 6:311–21.
7 Van der Woude HJ, Verstraete KL, Hogendoorn PCW, Taminiau AHM, Bloem JL. Fast dynamic contrast-enhanced substraction MR imaging in patients with musculoskeletal tumors.
8 Lucas JD, O'Doherty MJ, Cronin BF, *et al.* Prospective evaluation of soft tissue masses and sarcomas using fluorodeoxyglucose positron emission tomography. *Br J Surg* 1999; 86:550–6.
9 Lucas JD, O'Doherty MJ, Wong JC, *et al.* Evaluation of fluorodeoxyglucose positron emission tomography in the management of soft-tissue sarcomas. *J Bone Joint Surg [Br]* 1998; 80:441–7.
10 Jones J, Ross E, Matz L, Edwards D, Davies D. Retroperitoneal fibrosis. *Am J Med* 1970; 48:203–8.
11 Osborne CA, Butler J, Bloustein P, Sumner G. Idiopathic retroperitoneal fibrosis (sclerosing retroperitonitis). *Hum Pathol* 1987; 18:735–9.
12 Weiss SW. Proliferative fibroblastic lesions. From hyperplasia to neoplasia. *Am J Surg Pathol* 1986; 10(suppl 1):14–25.
13 Haggitt RC, Reid BJ. Hereditary gastrointestinal polyposis syndromes. *Am J Surg Pathol* 1986; 10:871–87.
14 Parsons M, Harris S, Longstaff A, Grainger R. Xanthogranulomatous pyelonephritis. A pathological, clinical and aetiological analysis of 87 cases. *Diagn Histopathol* 1983; 6:203–19.
15 Tomera K, Farrow G, Lieber M. Sarcomatoid renal carcinoma. *J Urol* 1983; 130:657–9.
16 Gonzalez-Cruss1 F. Retroperitoneal teratoma. *Pediatr Pathol* 1990; 10:1–36.
17 Lack EE, Travis W, Welch K. Retroperitoneal germ cell tumors in childhood. A clinical and pathological study of 11 cases. *Cancer* 1985; 56:602–8.
18 Graadt van Roggen JF, Welvaart K, de Roos A, Offerhaus GJA, Hogendoorn PCW. Adenocarcinoma arising within a tailgut cyst: clinicopathological description and follow-up of an unusual case. *J Clin Pathol* 1999; 52:310–2.
19 Meis JM, Enzinger FM. Myolipoma of soft tissue. *Am J Surg Pathol* 1991; 15:121–5.
20 Allen PW. *Tumors and proliferations of adipose tissue: a clinicopathologic approach.* New York: Masson Publishing, 1981.
21 Weiss SW. Lipomatous Tumours. *Monogr Pathol* 1996; 38:207–39.
22 Mentzel T, Fletcher CDM. Lipomatous tumours of soft tissues: an update. *Virchows Arch [A]* 1995; 427:353–63.
23 Dei Tos AP. Adipocytic tumors: new entities and evolving concepts. *Rev Esp Pathol* 1999; 32:271–3.
24 Lucas J, Nascimento AG, Sanjay KSS, Rock MG. Well-differentiated liposarcoma: the Mayo Clinic experience with 58 cases. *Am J Clin Pathol* 1994; 102:677–83.
25 Elgar F, Goldblum JR. Well–differentiated liposarcoma of the retroperitoneum: a clinicopathologic analysis of 20 cases, with particular attention to the extent of low-grade dedifferentiation. *Mod Pathol* 1997; 10:113–20.
26 Fletcher JA. Enzinger FM, Weiss SW, editors. *Soft tissue tumors*, 3rd edn. St. Louis, MO: Mosby; 1995;*5, Cytogenetic analysis of soft tissue tumors*, pp. 105–18.
27 Fletcher CD, Akerman M, Dal Cin P, *et al.* Correlation between clinicopathological features and karyotype in lipomatous tumors. A report of 178 cases from the CHAMP study group. *Am J Pathol* 1996; 148:623–30.
28 Mentzel T, Fletcher CD. Dedifferentiated myxoid liposarcoma: a clinicopathologic study suggesting a closer relationship between myxoid and well-differentiated liposarcoma. *Histopathol* 1997; 30:457–63.
29 Hashimoto H, Enjoji M. Liposarcoma. A clinicopathologic subtyping of 52 cases. *Acta Pathol Jpn* 1982; 32:933–48.
30 Weiss SW. Weiss SW, Brooks JSJ, editors. *Soft tissue tumors*. Augusta: Williams and Wilkins; 1996; *7, Lipomatous tumors*, pp. 207–39.
31 Henricks WH, Chu Y-C, Goldblum JR, Weiss SW. Dedifferentiated liposarcoma: a clinicopathological analysis of 155 cases with a proposal for an expanded definition of dedifferentiation. *Am J Surg Pathol* 1997; 21:271–81.
32 Weiss SW, Rao VK. Well differentiated liposarcoma (atypical lipoma) of deep soft tissue of the extremeties, retroperitoneum, and miscellaneous sites: a follow up study of 92 cases with analysis of the incidence of "dedifferentiation". *Am J Surg Pathol* 1992; 16:1051–68.
33 Kilpatrick SE, Doyon J, Choong PFM, Sim FH, Nascimento AG. The clinicopathologic spectrum of myxoid and round cell liposarcoma. A study of 95 cases. *Cancer* 1996; 77:1450–8.
34 Weiss SW, Rao VK. Well-differentiated liposarcoma (atypical lipoma) of deep soft tissues of the extremeties, retroperitoneum, and miscellaneous sites. A follow-up study of 92 cases with analysis of the incidence of "dedifferentiation". *Am J Surg Pathol* 1992; 16:1051–8.
35 Smith TA, Easley KA, Goldblum JR. Myxoid/round cell liposarcoma of the extremeties. A clinicopathologic study of 29 cases with particular attention to extent of round cell liposarcoma. *Am J Surg Pathol* 1996; 20:171–80.
36 Fletcher CDM. Will we ever reliably predict prognosis in a patient with myxoid and round cell liposarcoma? *Adv Anat Pathol* 1997; 4:108–13.
37 Abe S, Imamura T, Park P, *et al.* Small round-cell type of malignant peripheral nerve sheath tumor. *Mod Pathol* 1998; 11:747–53.

38 Erlandson RA, Klimstra DS, Woodruff JM. Subclassification of gastrointestinal stromal tumors based on evaluation by electron microscopy and immunohistochemistry. *Ultrastruct Pathol* 1996; 20:373–93.

39 Chan JKC. Mesenchymal tumors of the gastrointestinal tract: a paradise for acronyms (STUMP, GIST, GANT, and now GIPACT), Implications of c-kit in genesis, and yet another of many emerging roles of the interstitial cell of Cajal in the pathogenesis of gastrointestinal disease. *Adv Anat Pathol* 1999; 6:19–40.

40 Royani B, Smith TA, Reith JD, Goldblum JR. Retroperitoneal leiomyosarcomas unassociated with the gastrointestinal tract: a clinicopathologic analysis of 117 cases. *Mod Pathol* 1999; 12:21–8.

41 Sreekantaiah C, Davis JR, Sandberg AA. Chromosomal abnormalities in leiomyosarcomas. *Am J Surg Pathol* 1993; 142:293–305.

42 Kodet R, Newton WA, Jr, Hamoudi AB, Asmar L, Jacobs DL, Maurer HM. Childhood rhabdomyosarcoma with anaplastic (pleomorphic) features. A report of the intergroup rhabdomyosarcoma study. *Am J Surg Pathol* 1993; 17:443–553.

43 Huang CJ. Rhabdomyosarcoma involving the genitourinary organs, retroperitoneum and pelvis. *J Pediatr Surg* 1986; 21:101–7.

44 Kelly KM, Womer RB, Sorensen PHB, Xiong Q, Barr FG. Common and variant gene fusions predict distinct clinical phenotypes in rhabdomyosarcoma. *J Clin Oncol* 1997; 15:1831–6.

45 Barr FG, Galli N, Hollick J, Biegel JA, Rovera G, Emanuel B.S. Rearrangement of the PAX3 paired box in the pediatric solid tumor alveolar rhabdomyosarcoma. *Nature Gen* 1993; 3:113–7.

46 Davis RJ, D'Cruz CM, Lovell MA, Biegel JA, Barr FG. Fusion of PAX7 to FKHR by the variant t(1; 13)(p36; q14) translocation in alveolar rhabdomyosarcoma. *Cancer Res* 1994; 54:2869–72.

47 Regan JF, Juler GL, Schmutzer KJ. Retroperitoneal neurilemoma. *Am J Surg* 1977; 134:140–5.

48 White W, Shiu M, Rosenblum M, Erlandson R, Woodruff J. Cellular schwannoma: a clinicopathologic study of 57 patients and 58 tumors. *Cancer* 1990; 66:1266–75.

49 Casadei G, Scheithauer B, Hirose T, Manfrini M, van Houten C, Wood M. Cellular schwannoma: a clinicopathologic, DNA flow cytometric, and proliferation marker study of 70 patients. *Cancer* 1995; 75:1109–19.

50 Lodding P, Kindblom L, Angervall L, Stenman G. Cellular schwannoma. A clinicopathologic study of 29 cases. *Virchows Arch [A]* 1990; 416:237–48.

51 Woodruff JM. The pathology and treatment of peripheral nerve tumors and tumor-like conditions. *A Cancer J Clin* 1993; 43:290–308.

52 Ducatman BS, Scheithauer BW, Piepgras DG, Reiman HM, Ilstrup DM. Malignant peripheral nerve sheath tumors. A clinicopathologic study of 120 cases. *Cancer* 1986; 57:2006–21.

53 Brooks JSJ, Freeman M, Enterline HT. Malignant triton tumors. Natural history and immunohistochemistry of nine new cases with literature review. *Cancer* 1985; 55:2543–9.

54 Fletcher CDM. Pleomorphic malignant fibrous histiocytoma: fact or fiction? A critical reappraisal based on 159 tumors diagnosed as pleomorphic sarcoma. *Am J Surg Pathol* 1992; 16:213–28.

55 OrdóÄez NG. Desmoplastic small round cell tumor. *Am J Surg Pathol* 1998; 22:1303–13.

56 Gerald WL, Rosai J, Ladanyi M. Characterisation of the genomic breakpoint and chimeric transcripts in the EWS-WT1 gene fusion of desmoplastic small round cell tumor. *Proc Natl Acad Sci USA* 1995; 92:1028–32.

57 Kubben FJGM, Kroon FP, Hogendoorn PCW, *et al.* Absence of Epstein–Barr virus in a gastrointestinal stromal cell tumour (GIST) in an adult Human Immunodeficiency Virus-seropositive patient with past Epstein–Barr virus (EBV) infection. *Eur J Gastroent Hepatol* 1997; 9:721–4.

58 Hirota S, Isozaki K, Moriyama Y, *et al.* Gain-of-function mutations in c-kit in human gastrointestinal stromal tumors. *Science* 1998; 279:577–80.

59 Tsang WYW, Chan JKC. Kaposi-like infantile hemangioendothelioma. A distinctive vascular tumor of the retroperitoneum. *Am J Surg Pathol* 1991; 15:982–9.

60 Vallat-Decouvelaere A, Dry S, Fletcher CDM. Atypical and malignant solitary fibrous tumors in extrathoracic locations. Evidence of their comparability to intrathoracic tumors. *Am J Surg Pathol* 1998; 22:1501–11.

61 Luttges J, Mentzel T, Hubner G, Kloppel G. Solitary fibrous tumor of the pancreas: a new member of the small group of mesenchymal pancreatic tumours. *Virchows Arch [B]* 1999; 435:37–42.

62 Fletcher CDM. Haemangiopericytoma—a dying breed? Reappraisal of an 'entity' and its variants: a hypothesis. *Curr Diagn Pathol* 1994; 1:19–23.

63 Nappi O, Ritter JH, Pettinato G, Wick MR. Hemangiopericytoma: histopathological pattern or clinicopathologic entity? *Semin Diagn Pathol* 1995; 12:221–32.

64 Lack EE, Cubilla AL, Woodruff JM, Lieberman PH. Extra-adrenal paragangliomas of the retroperitoneum: a clinicopathologic study of 12 tumors. *Am J Surg Pathol* 1980; 4:109–20.

65 Moran CA, Albores-Saavedra J, Wenig BM, Mena H. Pigmented extraadrenal paragangliomas. A clinicopathologic and immunohistochemical study of five cases. *Cancer* 1997; 79:398–402.

66 Kransdorf MJ. Benign soft-tissue tumors in a large referral population: distribution of specific diagnoses by age, sex, and location. *Am J Roentgenol* 1995; 164:395–402.

67 Meis JM, Enzinger FM. Inflammatory fibrosarcoma of the mesentery and retroperitoneum. A tumor closely simulating inflammatory pseudotumor. *Am J Surg Pathol* 1991; 15:1146–56.

68 Shmookler BM. Retroperitoneal synovial sarcoma. A report of four cases. *Am J Clin Pathol* 1982; 77:686–91.

69 Miracco C, Laurini L, Santopietro R, *et al.* Intimal-type primary sarcoma of the aorta. Report of a case with evidence of rhabdomyoblastic differentiation. *Virchows Arch [B]* 1999; 435:62–6.

70 Newman PL, Fletcher CDM. Malignant mesenchymoma. Clinicopathologic analysis of a series with evidence of low-grade behaviour. *Am J Surg Pathol* 1991; 15:607–14.

71 Gurarangan S, Bowman LC, Parham DM, *et al.* Primary extrarenal rhabdoid tumors. Clinicopathologic features and response to ifosfamide. *Cancer* 1993; 71:2653–9.

72 Shimada H, Newton WA, Jr, Soule EH, Qualman SJ, Aoyama C, Maurer HM. Pathologic features of extraosseous Ewing's sarcoma: a report from the intergroup rhabdomyosarcoma study. *Hum Pathol* 1988; 19:442–53.

73 Bane B, Evans HL, Ro JY, *et al.* Extraskeletal osteosarcoma. A clinicopathologic review of 26 cases. *Cancer* 1990; 65:2762–70.

74 van Rijswijk CSP, Hogendoorn PCW, Tjong A Lieng JGS, Kroon HM. Retroperitoneal extraskelatal osteosarcoma. *J Clin Pathol* 2000.

75 Akerman M, Willen H. Critical review on the role of fine needle aspiration in soft tissue tumors. *Pathol Case Rev* 1998; 3:111–7.

76 Coindre JM, Trojani M, Contesso G, *et al.* Reproducibility of a histopathologic grading system for adult soft tissue sarcoma. *Cancer* 1986; 58:306–9.
77 Costa J, Wesley RA, Glatstein E, Rosenberg SA. The grading of soft tissue sarcomas. Results of a clinicohistopathologic correlation in a series of 163 cases. *Cancer* 1984; 53:530–41.
78 Meis-Kindblom JM. On the comparison of apples, oranges and sundry fruits: problems with grading and prognostication in soft tissue tumors. *Rev Esp Pathol* 1999; 32:426–7.
79 Enneking WF, Spanier SS, Goodman MA. A system for the surgical staging of musculoskeletal sarcoma. *Clin Orthop* 1980; 153:106–20.
80 Enneking WF. Musculoskeletal tumor staging: 1988 update. *Cancer Treat Res* 1989; 44:39–49.
81 Enneking WF. Enneking WF, editors. *Clinical musculoskeletal pathology*, 3rd edn. Gainesville, Florida: University Presses of Florida; 1990; *Appendix A. The staging system for benign and malignant tumors of the musculoskeletal system*, pp. 451–66.
82 Fletcher CDM, Kempson RL, Weiss SW. Recommendations for the reporting of soft tissue sarcoma. *Virchows Arch [B]* 1999; 434:187–91.
